# Primary histiocytic sarcoma of the central nervous system: a case report with platelet derived growth factor receptor mutation and PD-L1/PD-L2 expression and literature review

**DOI:** 10.1186/s13014-018-1115-x

**Published:** 2018-09-05

**Authors:** Jackson M. May, Mark R. Waddle, Daniel H. Miller, William C. Stross, Tasneem A. Kaleem, Byron C. May, Robert C. Miller, Liuyan Jiang, Gerald W. Strong, Daniel M. Trifiletti, Kaisorn L. Chaichana, Ronald Reimer, Han W. Tun, Jennifer L. Peterson

**Affiliations:** 1 Department of Radiation Oncology, Mayo Clinic, 4500 San Pablo Road South, Jacksonville, FL 32224 USA; 2 Department of Neurological Surgery, Mayo Clinic, Jacksonville, Florida, USA; 3 Department of Pathology and Laboratory Medicine, Mayo Clinic, Jacksonville, Florida, USA; 4 Department of Hematology and Medical Oncology, Mayo Clinic, Jacksonville, Florida, USA

**Keywords:** Histiocytic sarcoma, CNS, Radiation, Review

## Abstract

**Background:**

Histiocytic sarcoma (HS) is an aggressive malignant neoplasm. HS in the central nervous system is exceptionally rare and associated with a poor prognosis. This report documents a case of primary HS of the central nervous system with treatment including surgery, radiotherapy, and chemotherapy.

**Case presentation:**

Our patient was a 47 year old female presenting with progressive ataxia, headaches, imbalance, nausea, vomiting, and diplopia. MRI showed a heterogeneously enhancing lesion approximately 2.9 × 3.0 × 2.3 cm centered upon the cerebellar vermis with mild surrounding vasogenic edema and abnormal enhancement of multiple cranial nerves. The patient underwent surgical debulking, which revealed histiocytic sarcoma with grossly purulent drainage. Staging revealed diffuse leptomeningeal involvement, primarily involving the brain and lower thoracic and lumbar spine. She underwent adjuvant radiotherapy to the brain and lower spine and was started on high dose methotrexate. However, she experienced progressive disease in the cervical and thoracic spine as well as pulmonary involvement. Genomic sequencing of her tumor showed a mutation in the platelet-derived growth factor receptor A (p.V0681) which could be targeted with Dasatinib. However, she did not tolerate Dasatinib and she succumbed to progressive disseminated disease eight months from original diagnosis. Our pathologic evaluation also revealed expression of PD-L1 and PD-L2 by tumor cells raising the potential therapeutic role for immune checkpoint inhibition.

**Conclusions:**

This case provides an example of effective CNS control with resection and moderate doses of radiation therapy. A review of the literature confirms aggressive multidisciplinary treatment is the most effective treatment against this disease. In addition, genomic sequencing may play an important role in determining new therapeutic options. However, CNS histiocytic sarcoma remains an aggressive disease with a propensity for early widespread dissemination and few long term survivors.

## Background

Histiocytic sarcoma (HS) is an uncommon malignant neoplasm with morphological and immunophenotypic features of histiocytic tissue. HS presents most commonly with a mass involving soft tissues, gastrointestinal tract, and/or lymph nodes [16]. Primary histiocytic sarcoma of the central nervous system (CNS) is extremely rare and accounts for less than 1% of all lymphohematopoietic neoplasms [22]. A typical finding with CNS HS is the presence of an inflammatory infiltrate and the abundance of inflammatory cells can cause neoplastic cells to be overlooked and thus a misdiagnosis may occur [2]. Many previous cases are thought to be misdiagnosed due to lack of genetic markers, but the disease can now be recognized by its biological markers, such as positive expression of CD 163, CD 68, and lysozyme, which allow differentiation between CNS HS and other hematopoietic neoplasms [5], such as B-cell or T-cell non-Hodgkin’s Lymphoma [16, 21]. While the optimal treatment for CNS HS is not well defined, surgery is the primary modality with radiotherapy and chemotherapy frequently used as adjuvant treatments. Overall, CNS HS carries a poor prognosis, but there have been reported cases of long term disease free survival in adults when multidisciplinary treatment of radiation therapy, chemotherapy, and surgery are applied [2, 11, 23]. Here, we present a case of primary CNS HS with leptomeningeal spread treated with tri-modality therapy and provide a review of the available literature for this aggressive disease.

## Case presentation

**Fig. 1:**
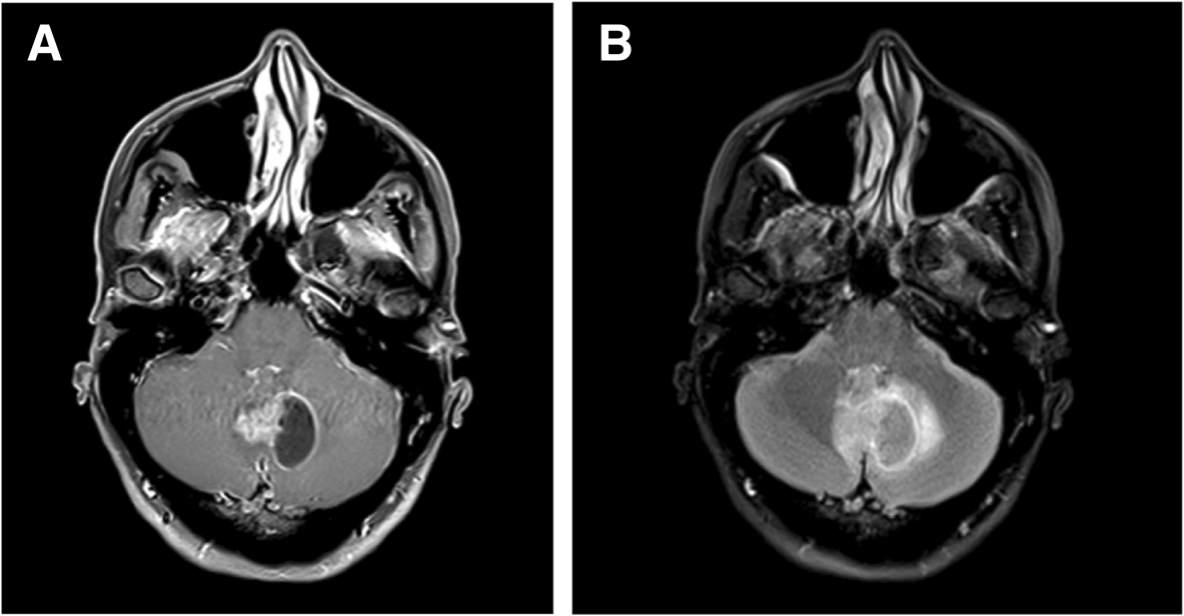
**a**) Axial T1 Post Gadolinium Fat Saturation. **b**) T2 FLAIR Fat Saturation

Our patient was a 47 year old female with a history of ulcerative colitis, Sjogren’s syndrome, migraines, and fibromyalgia who presented with a 6 month history of left lower extremity paresthesia followed by a 2 month history of progressive headaches, imbalance, ataxia, nausea, vomiting, and diplopia. Neurologic examination revealed severe gait ataxia requiring assistance to stand or walk and nystagmus with lateral gaze. MRI revealed a 3.0 cm heterogeneously enhancing mixed cystic and solid mass centered upon the cerebellar vermis with mild surrounding vasogenic edema and abnormal thickened enhancement of the bilateral vestibular nerves, left facial nerve, and right trigeminal nerve (Fig. 1).

**Fig. 2:**
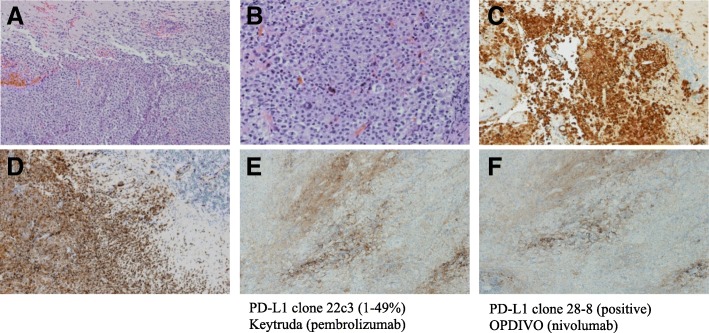
Final Pathological Examination. The H&E section show the diffuse proliferation of neoplastic histiocytes invading normal brain parenchyma (**a** × 10, upper normal brain; bottom neoplastic histiocytes); the neoplastic histiocytes show marked cytological atypia with brisk mitosis (**b** × 20). Immunohistochemical studies show the histiocytes positive for CD 163 (**c** ×10), CD68 (**d** × 10), CD45 and vimentin; negative for CD20, CD3, CD30, s-100, CD1a, CD21, CD23, pancytokeratin, MPO, CD61, CD123, GFAP, and BRAF. The neoplastic histiocytes are also positive for PD-L1 with both clones (**e**× 10, 22C3 and **f**× 10, 28–8)

The patient was promptly started on steroids, admitted to the hospital, and underwent a midline suboccipital craniotomy for debulking of the large intracerebellar tumor. Upon entering the cystic cavity, grossly purulent material without hemorrhage was noted. Gram stain and cultures were negative for infection. A subtotal resection was achieved and final pathology rendered the diagnosis of primary CNS histiocytic sarcoma (see Fig. 2). The H&E section (Fig. 2a and b) revealed sheets of large neoplastic cells with marked cytological atypia, brisk mitosis with occasional multilobated nuclei, and focal necrosis. Extensive immunohistochemical studies (Fig. 2c and d) showed the neoplastic cells positive for CD163, CD68, CD45, and Vimentin; negative for CD20, CD3, CD30, s-100, CD1a, CD21, CD23, pancytokeratin, MPO, CD61, CD123, GFAP, and BRAF. Further immunostains for PD-L1 with two different antibody clones (22C3 and 28–8) were also performed and showed more than 50% of the tumor cells were positive with membrane stain (Fig. 2e and f).

**Fig. 3:**
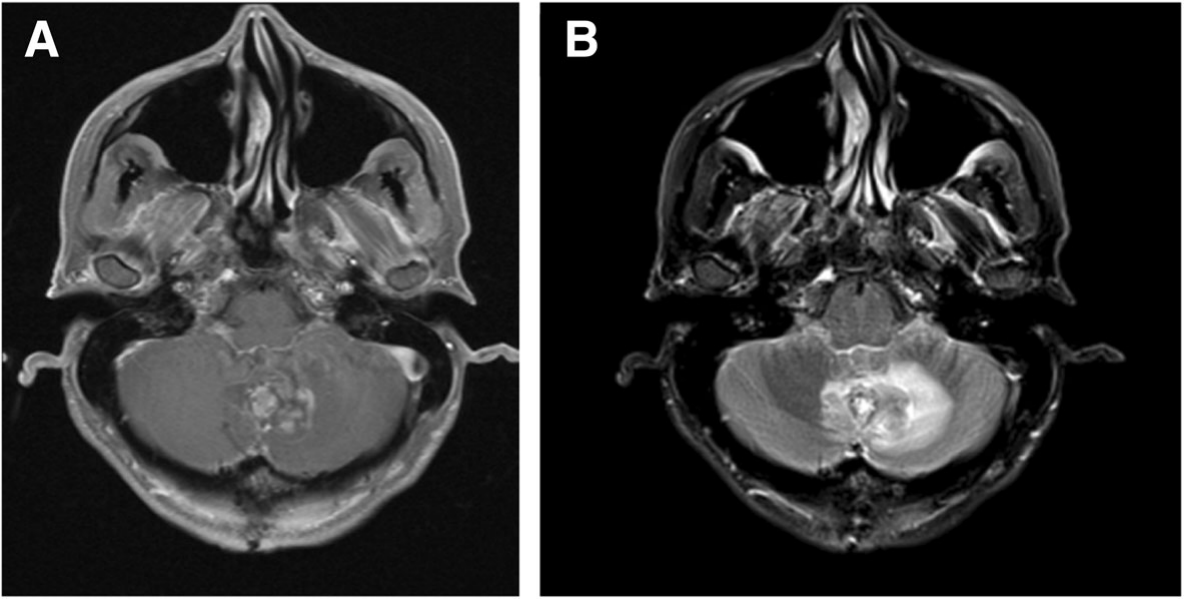
**a**) Axial T1 Post Gadolinium Fat Saturation. **b**) T2 FLAIR Fat Saturation

Post-operative MRI showed a midline posterior occipital craniotomy with subtotal resection of tumor and continued bilateral-enhancement along multiple cranial nerves, concerning for leptomeningeal spread (Fig. 3).

**Fig. 4:**
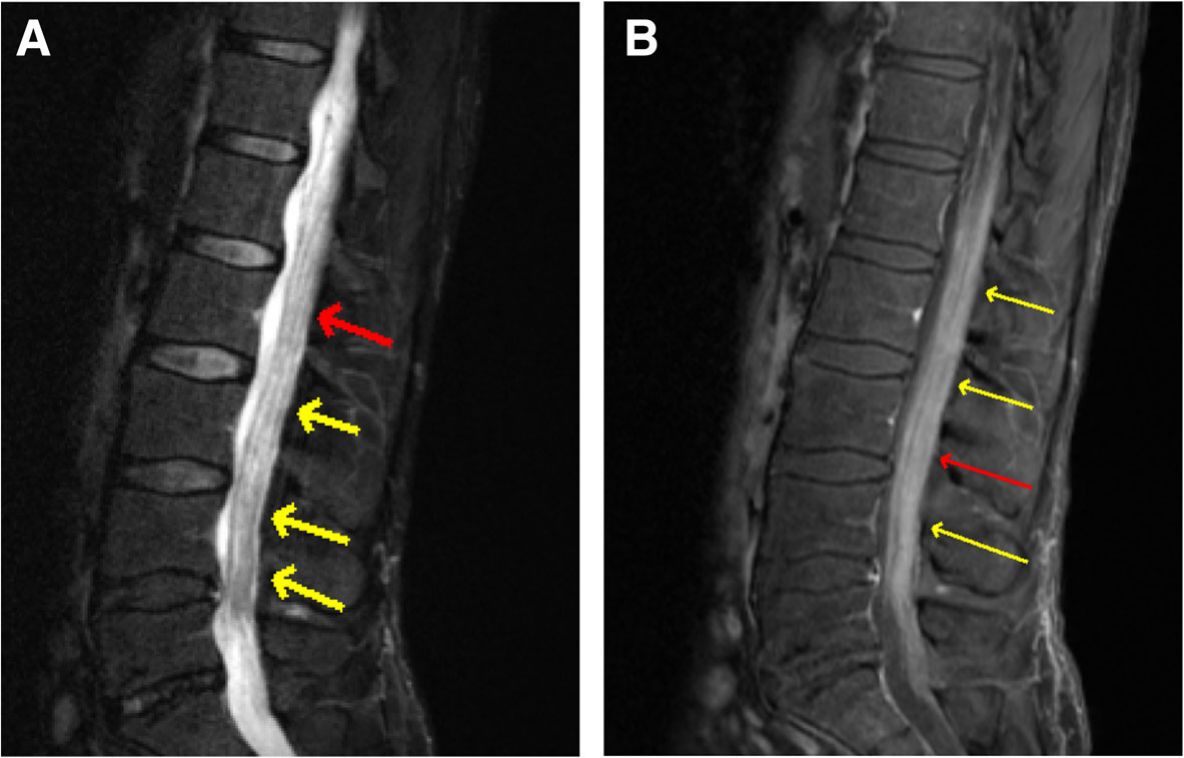
**a**) Sagittal T2. **b**) Sagittal T1 Post Contrast

A staging PET scan was performed two weeks following surgery suggesting diffuse leptomeningeal spread. A complete spinal MRI confirmed diffuse leptomeningeal spread in the lower thoracic and lumbar spine as well as cauda equina involvement. Due to the convincing evidence on MRI, a CSF analysis was deferred. Standard lab work was within limits, including LDH (Fig. 4).

Three weeks following surgery, due to progressive lower back pain, she initiated radiation therapy including whole brain radiotherapy (30.6 Gy in 17 fractions) followed by a posterior fossa boost (5.4 Gy in 3 fractions) and a gross tumor boost (9 Gy in 5 fractions), for a total dose of 45 Gy in 25 fractions to gross disease with simultaneous radiation therapy to the lower thoracic and lumbar spine, receiving 40 Gy in 20 fractions, encompassing the areas of leptomeningeal disease in the spine and cauda equina. Full craniospinal radiotherapy was not performed to limit profound cytopenias that may have prevented further cytotoxic chemotherapy.

Interval follow up MRI showed a partial response with persistent posterior fossa disease but near complete resolution of previous leptomeningeal enhancement. Our plan was to initiate chemotherapy with a primary CNS lymphoma regimen with high CNS penetrating therapeutic agents including high-dose methotrexate, high-dose Ara-C, and thiotepa followed by high-dose chemotherapy with BCNU and thiotepa rescued by autologous stem cell transplantation. The patient was started on high dose intravenous methotrexate (HD-MTX) therapy, but received only one cycle secondary to patient intolerance.

A CT of the chest approximately two weeks after initiation of HD-MTX therapy for left sided rib pain showed new pulmonary nodules and a left sided pleural effusion, suspicious for metastatic disease. Thoracentesis revealed findings consistent with a malignant pleural effusion. The patient was initiated on cladribine (Leustatin).

Within two weeks after the completion of this infusion, the patient developed headaches, somnolence, fever, and nuchal rigidity. Malignant meningitis was suspected and the patient was started on IV antibiotics. Lumbar puncture was recommended but the patient declined. Her mental status significantly improved following antibiotic treatment. Brain MRI showed continued response of the cerebellar residual disease and no new intra-cranial lesions, but an MRI of the cervical spine showed new lesions consistent with metastatic disease in the untreated cervical spinal cord and thoracic vertebrae, including intramedullary involvement. She underwent further radiation therapy targeting C4-C6 and T1-T7 (20 Gy in 5 fractions). The patient rapidly showed improvement in symptoms.

Genomic sequencing of her tumor showed a novel mutation in the platelet-derived growth factor receptor A (p.V608I) and therefore, she was started on Dasatinib, a tyrosine kinase inhibitor (TKI) with known CNS penetration [24]. Unfortunately, this medication was poorly tolerated because of nausea, diarrhea, and acute pancreatitis so it was discontinued after one week.

The patient was readmitted to the hospital for continued decline in functional status, weakness, and failure to thrive. CT images of the chest, abdomen, and pelvis showed progressive bilateral pulmonary nodules. An MRI of the spine showed persistent but significantly improved leptomeningeal enhancement. Due to progression of symptoms, worsening systemic disease despite control of CNS disease, and limited systemic options available, the patient chose to enroll in hospice. The patient expired four weeks later, eight months after initial diagnosis.

## Discussion

Histiocytic sarcoma of the CNS is an uncommon diagnosis with only about 30 total cases reported in the literature. The median age for diagnosis of HS is 46 years, which is equally distributed in males and females [20]. The prognosis for HS of the CNS is extremely poor with a median survival of 4.5 months, the longest recurrence free period reported being 42 months [6, 23].

This case was unique and merits discussion for several reasons. First, at the time of surgery, entry into the cerebellar lesion was associated with drainage of purulent material, which has been documented previously in only three other cases [1, 3, 26]. This was an unexpected finding at the time of surgery and no evidence of infection was identified. Second, this patient had extensive and symptomatic leptomeningeal disease with cranial nerve and spinal involvement by MRI. Leptomeningeal disease is relatively common in CNS HS, 10/31 (32%) documented cases had leptomeningeal involvement, which is associated with a poor quality of life and poor survival. In our case, this disease was treated aggressively with moderate dose radiation therapy, 45 Gy to the gross tumor in the cerebellum and 40 Gy to the leptomeningeal disease in the spine, which did achieve continued disease control, even 6 months after treatment. This raises the question as to whether patients with leptomeningeal spread would benefit from craniospinal radiotherapy. However, full craniospinal radiotherapy can induce profound cytopenias that could limit or delay the use of systemic therapy which has known benefit for histiocytic sarcoma. The other important aspect of this case is that we were able to obtain adequate amount of tissue from surgical resection of the brain tumor and performed genomic sequencing revealing a novel PDGFR mutation which is highly targetable with TKI’s. There is very limited literature regarding genomic data in CNS histiocytic sarcoma. One additional case report describes a patient with CNS histiocytic sarcoma with genomic sequencing revealing BRAF V600E mutation. The patient experienced a dramatic clinical and radiographic response with vemurafenib, though the response was not durable and the patient expired 6 months after initiation of treatment [17]. Our pathologic evaluation showed PD-L1 and PD-L2 expression by the tumor, which suggests that immunotherapy with checkpoint inhibitors should be explored. Using inhibitors to suppress PD-L1/PD-1 interaction has shown promising effects for treatment of various advanced cancers, but no literature is available for HS of the CNS [12]. Overexpression of PD-1 in an animal model has suggested antitumor immunity may be suppressed in histiocytic sarcoma, although this is yet to be shown in humans [27]. To the best of our knowledge, our case is the first report of PD-L1/PD-L2 expression in histiocytic sarcoma of the CNS.

A review of the literature for CNS HS was performed and the identified cases are shown in Table 1. Only three of thirty patients (10%) were alive with no evidence of disease at the time of publication, 16, 23, and 42 months after presentation [2, 11, 23]. Treatment for these cases consisted of surgery followed by concurrent chemoradiation with temozolomide and high dose focal radiation. In the two cases with radiation dose reported, the total dose given was 54 Gy and 61.4 Gy. This is consistent with the findings in our case that moderate doses of radiation are needed for local control.

**Table 1:** Reported cases of Primary Histiocytic Sarcoma of the CNS and their characteristics

Author & Year	Age, Gender	Site(s)	Number of Lesions	Size (cm)	Primary Treatment	Outcome
^a^Gill-Samra et al. 2012 [14]	38 yr./F	Temporal	Multiple	5	Surgery+ WBRT: 45Gy in 25 fractions+ Chemotherapy	DOD 3 weeks after presentation
^a^Toshkezi et al. 2010 [29]	71 yr./F	Spine and leptomeninges	Solitary	2.5x1x 1.1	Surgery+ RT: 44 Gy in 22 fractions	DOD 5 months after presentation
^a^Bell et al. 2012 [3]	62 yr./F	Cerebellum	Solitary	ND	Surgery	AWPD 24 months
^a^Bell et al. 2012 [12]	34 yr./M	Frontal	Solitary	2	Surgery	AWPD 10 months
^a^ Devic et al., 2012 [10]	43 yr./F	Corpus callosum, Cerebellum, and Spine	Multiple	ND	Chemotherapy	DOD 10 months after presentation
^a^Wang et al., 2012 [31]	55 yr./F	Corpus callosum	Multiple	ND	Surgery+3D CRT: 16 Gy	DOD 4 months after presentation
^a^Torres et al., 1996 [28]	20 mo/M	Leptomeninges	Solitary	ND	Chemotherapy	DOD 3 months after presentation
^a^ Cheuk et al., 2001 [9]	69 yr./F	Parietal	Solitary	1.5	Surgery+ WBRT+ Chemotherapy	DOD 8 months after presentation
^a^ Cheuk et al., 2001 [9]	43 yr./M	Spine	Solitary	1.7	Surgery+ WBRT+ Chemotherapy	AWPD at 5 months
^a^ Cheuk et al., 2001 [21]	11 yr./M	Cerebellum, occipital, and frontal	Multiple	.7–1	Surgery	DOD 4 months after presentation
^a^ Sun et al., 2003 [26]	13 yr./M	Occipital and leptomeninges	ND	ND 1.1	Surgery	DOD 7 months after presentation
^a^Cao et al., 2007 [6]	53 yr./F	Cavernous sinus, relapsed to mediastinum	Solitary	3.1 × 2.9 × 2.2	Surgery+ RT 54 Gy	DOD 4 years after presentation
^a^Almefty et al., 2013 [1]	16 yr./M	Parietal	Solitary	3.5 × 4.4 × 4.0	Surgery+ IMRT: 60 Gy in 30 fractions	DOD 4 months after presentation
^a^Wu et al., 2013 [32]	50 yr./M	Parieto-occipital	Solitary	1.7	Surgery+ SRS: 30 Gy in 5 fractions	AWPD at 18 months
^a^ Laviv et al., 2013 [19]	58 yr./M	Frontal	Solitary	6.5	Surgery	DOD 4 months after presentation
^a^Gomi et al.. 2012 [15]	17 mo/F	Cerebellum, dissemination to spine	Solitary	4.7 × 4.3 × 4.3	Surgery+ Chemotherapy	AWPD at 16 months
^a^Gentzler et al., 2011 [13]	52 yr./F	Parietal	Solitary	1.7	Palliative Care	Expired from lung cancer
^a^ Perez-Ruiz et al., 2013 [23]	41 yr./F	Temporal and leptomeninges	Solitary	1.5 × 2	Surgery+ TMZ + IMRT: 61.2Gy	ANED 42 months
^a^Chalasani et al., 2013 [7]	44 yr./M	Corpus callosum	Multiple	3.5 and 2.6	Chemotherapy+ WBRT: 26 Gy + Boost to 46 Gy	DOD 27 weeks after presentation
^a^Idbaih et al., 2014 [17]	40 yr./M	Temporal	Solitary	ND	Chemotherapy	DOD 6 months after presentation
^a^Moulinger et al., 2014 [22]	63 yr./F	Pons	Multiple	ND	Chemotherapy	DOD 20 days after completion of treatment
^a^ Bai et al., 2014 [2]	52 yr./M	Frontal	Solitary	ND	Surgery+ TMZ + IMRT: 54 Gy	ANED 16 months
^a^Foster et al., 2015 [11]	15 yr./F	Frontal	Solitary	5.8 × 4.7 × 4.0	Surgery+ TMZ + 3D CRT	ANED 23 months
^a^ Chen et al., 2015 [8]	61 yr./M	Meckel’s Cave	Solitary	1.5 × 1.1 × 1.8	Radiotherapy 66Gy in 33 fractions + 1 cycle CVP + 2 cycles CHOP	AWPD 31 months
^a^Brown et al., 2015 [4]	23 yr./M	Cerebellopontine	Solitary	6	Chemotherapy+ IMRT	AWPD 60 months
^a^So et al., 2015 [25]	59 yr./M	Parietal, Corpus callosum, Frontal and Spine	Multiple	ND	Chemotherapy	DOD 8 months after presentation
Zanelli et al., 2017 [33]	45 yr./F	Leptomeninges	N/A	N/A	No therapy	DOD 2 months after presentation
^a^ Ueno et al., 2016 [30]	65 yr./M	Frontal, parietal, spine, and leptomeninges	Multiple	ND	Radiotherapy	AWPD 11 months
Kim et al., 2017 [18]	16 yr./M	Corpus callosum, frontoparietal	Solitary	6.5 × 5.3	Surgery+ Chemotherapy+ Radiotherapy 60Gy	DOD 12 months after presentation
Marguet et al., 2018 [21]	67 yr./M	Periventricular, hypothalamic and sellar region, leptomeningeal involvement.	Multiple	2.1, 1.4	No therapy	DOD 7 months after presentation
Present case	47 yr/ F	Cerebellar vermis and leptomeninges	Multiple	3.0 × 2.9 × 2.3	Surgery + WBRT: 30.6 Gy + Boost to 45 Gy + Chemotherapy	DOD 8 months after presentation

*ANED* Alive and no evidence of disease, *AWPD* Alive with progressive disease, *DOD* Dead of disease, *ND* Not Determined, *PTV* Planning target volume, *WBRT* Whole Brain Radiation Therapy, *3D CRT* 3D Conformal Radiotherapy, *IMRT* Intensity modulated radiotherapy, *TMZ* Temozolomide, *CVP* Cyclophosphamide, vincristine, and prednisone, *CHOP* Cyclophosphamide, doxorubicin, vincristine, and prednisone

^a^Indicates patients from manuscript Zanelli et al., 2017 [33]

The majority of cases in the literature received some form of surgery, radiation, and/or chemotherapy. The current standard treatment for CNS HS is surgery to achieve a maximal safe resection and for tissue diagnosis and relieving cerebral edema. An aggressive, multimodality approach is preferred with postoperative radiation therapy +/− chemotherapy, when the disease is unifocal. When the disease has become multifocal, further aggressive radiotherapy and combination chemotherapy is essential. Chemotherapeutics used previously include temozolomide (TMZ), cyclophosphamide, doxorubicin, vincristine and prednisone (CHOP), rituximab, ifosfamide, carboplatin, etoposide (ICE), methotrexate, and thalidomide. There are no documented cases in the literature of successful treatment of multifocal disease. Thus, HS is a very aggressive disease when found in the central nervous system which warrants aggressive treatment with multiple modalities. Despite limited long term survivals of this disease, there have been approximately 10% of cases with long term survival with aggressive treatment including surgery, dose escalated radiation therapy, and chemotherapy.

## Conclusion

In this case we present a patient with a primary histiocytic sarcoma of the CNS, an extremely rare diagnosis with a poor prognosis. This patient was treated aggressively with surgery, radiation therapy, chemotherapy, and targeted therapy informed by genomic sequencing but ultimately expired of her progressive disease approximately 8 months after diagnosis. The genomic sequencing of the tumor tissue showed a novel PDGFR mutation, which could be targeted with TKI’s. We showed expression of PD-L1 and PD-L2 by the tumor cells suggesting potential therapeutic role for immune checkpoint inhibitors. Our case demonstrates a durable response of CNS disease with radiation doses 40 Gy and above. Therefore, we recommend aggressive treatment with curative radiation doses to the CNS whenever appropriate and possible. Novel therapeutic agents such as TKI’s and checkpoint inhibitors need to be explored.

## Abbreviations

BCNU: Carmustine
CD: Cluster of differentiation
CHOP: Cyclophosphamide, Doxorubicin, Vincristine, and Prednisone
CNS: Central Nervous System
CSF: Cerebrospinal Fluid
CT: Computed Tomography
GFAP: Glial fibrillary acidic protein
Gy: Grey
H&E: Haemotoxylin and Eosin
HD-MTX: High Dose Methotrexate
HS: Histiocytic Sarcoma
ICE: Ifosfamide, Carboplatin, and Etoposide
IV: Intravenous
LDH: Lactate Dehydrogenase
MPO: Myeloperoxidase
MRI: Magnetic Resonance Imaging
PD-1: Programmed Cell Death Protein 1
PDGFR: Platelet Derived Growth Factor Receptor
PD-L1: Programmed Death-Ligand 1
PD-L2: Programmed Death Ligand-2
PET: Positron Emission Tomography
TKI: Tyrosine Kinase Inhibitor
TMZ: Temozolomide
